# Commensal-to-pathogen transition: One-single transposon insertion results in two pathoadaptive traits in *Escherichia coli* -macrophage interaction

**DOI:** 10.1038/s41598-017-04081-1

**Published:** 2017-07-03

**Authors:** João T. Proença, Duarte C. Barral, Isabel Gordo

**Affiliations:** 1 Instituto Gulbenkian de Ciência, R. Q.ta Grande 6, 2780-156 Oeiras, Portugal; 2 CEDOC, NOVA Medical School Faculdade de Ciências Médicas, Universidade NOVA de Lisboa, Lisboa, Portugal

## Abstract

*Escherichia coli* is both a harmless commensal in the intestines of many mammals, as well as a dangerous pathogen. The evolutionary paths taken by strains of this species in the commensal-to-pathogen transition are complex and can involve changes both in the core genome, as well in the pan-genome. One way to understand the likely paths that a commensal strain of *E*. *coli* takes when evolving pathogenicity is through experimentally evolving the strain under the selective pressures that it will have to withstand as a pathogen. Here, we report that a commensal strain, under continuous pressure from macrophages, recurrently acquired a transposable element insertion, which resulted in two key phenotypic changes: increased intracellular survival, through the delay of phagosome maturation and increased ability to escape macrophages. We further show that the acquisition of the pathoadaptive traits was accompanied by small but significant changes in the transcriptome of macrophages upon infection. These results show that under constant pressures from a key component of the host immune system, namely macrophage phagocytosis, commensal *E*. *coli* rapidly acquires pathoadaptive mutations that cause transcriptome changes associated to the host-microbe duet.

## Introduction

Most *Escherichia coli* are commensal bacteria that colonize the mammalian gastrointestinal tract soon after birth. However, *E*. *coli* is also a major cause of several diseases such as urinary tract infection, gastroenteritis and neonatal meningitis^1^. The evolutionary history of *E*. *coli* is marked by many events resulting from different processes: horizontal gene transfer (HGT), reorganization of genome structure and accumulation of mutations in the genome^2^. Despite the rich mechanisms of evolutionary change, robust phylogenetic groups can be determined, some of which enriched for pathogenic strains. While there is evidence that some pathogenic strains evolved from commensal *E*. *coli* strains^2–4^, it remains difficult to determine which genes or genetic changes are responsible for *E*. *coli* becoming pathogenic. This difficulty emerges from the many traits that distinguish the different pathogenic strains of *E*. *coli* from commensal strains, such as the ability to colonize a given mucosal site, evade host defenses and cause damage to host tissues.

The switch from commensal to pathogen is an important transition, which can involve acquisition of virulence genes by HGT and/or accumulation of pathoadaptive mutations^5, 6^. Remarkably, commensal *E*. *coli* such as K12 have the sufficient genetic tools to survive and replicate inside eukaryotic cells, since two mutations are sufficient for this switch in behaviour to occur^7^. Examples of pathoadaptive mutations in naturally-occurring pathogens include: the loss of *mucA* in *Pseudomonas aeruginosa*, which increases its ability to evade phagocytosis and resist pulmonary clearance^8^; the loss of *oprD* by *P*. *aeruginosa*, with an associated carbapenem-resistance phenotype, which results in increased levels of cytotoxicity against macrophages (MΦs) and increased colonization and dissemination to the spleen of mice^9^; polymorphism in *hopZ* in *Pseudomonas syringiae*, which allows for immune evasion in plants^10^; and allelic variation in FimH, the type 1 adhesin of *E*. *coli*, which can change the ability of uropathogenic strains to colonize and invade bladder tissue^11^.

Our understanding of how often and by which mechanisms bacteria transit from a commensal to a pathogenic lifestyle is still far from complete. Experimental evolution is a powerful methodology to study the emergence and evolution of pathogenic traits^12, 13^. Recently, we developed an experimental evolution setup to determine the emergence of possible pathogenic traits in commensal *E*. *coli*, by following its adaptation under an antagonistic interaction with one of the key sentinels of the innate immune system, MΦs^14^. With this approach we were able to observe the emergence of *E*. *coli* clones that evolved phenotypes that provide direct fitness advantage in the interaction with MΦs. Some of the evolved clones carry mutations caused by IS insertions at three different loci: the promoter region of *yrfF*, the coding region of *yiaW* and the coding region of *potD*. These clones form mucoid colonies, due to overproduction of colonic acid, show increased ability to evade MΦ phagocytosis *in vitro* and are also more virulent *in vivo* ^14^.

Upon phagocytosis, bacteria-containing phagosomes follow a maturation process whereby they progressively acidify and acquire hydrolytic enzymes. This occurs through fusion with organelles of the endocytic pathway, namely endosomes and lysosomes, leading to the formation of phagolysosomes^15^. Several pathogens have evolved adaptations to cope with the harsh environment created inside maturing phagosomes^16^. Most strategies involve the arrest of phagosomal maturation or the escape from phagosomes. For example, *Mycobacterium tuberculosis* (Mtb) is able to block the maturation of phagosomes, which retain the characteristics of early endosomes and exhibit limited acidification^17, 18^. It has also been reported that Mtb can escape phagolysosomes by translocating to the cytosol^19^. Similar to Mtb, *Legionella pneumophila*-containing phagosomes do not fully acidify because their fusion with lysosomes is inhibited^20, 21^. Within the Enterobacteriaceae family, *Shigella flexneri* is able to disrupt the phagosomal membrane and translocate to the cytoplasm, where it can replicate^22^. Furthermore, *Salmonella* arrests phagosome maturation at a late stage. In this case, the acidification of the phagosome is an environmental cue for the expression of pathogenicity island 2^23^, which is necessary for bacterial replication in this adverse environment^24^.

Autophagy is another pathway that plays an important role in the defence against intracellular pathogens. Autophagy is normally used by cells to degrade intracellular cytosolic constituents, including organelles. This pathway relies on the formation of a double membrane organelle, the autophagosome, which fuses with late endosomes and lysosomes to form degradative compartments. Autophagy can be used by host cells to eliminate Mtb, *Shigella* spp., *Listeria monocytogenes* and *Salmonella enterica*, among other bacteria and protozoa^25^. However, autophagy can also be subverted by intracellular pathogens. For example, *Staphylococcus aureus* prevents autophagosome maturation, escaping to the cytosol and replicating there^26^.

Here, we tested if the *E*. *coli* clones evolved under continuous selective pressure of MΦs acquired traits that could confer them a fitness advantage in the intracellular environment of MΦs. We found that MΦ-adapted clones display increased intracellular survival and delay phagosome maturation due to a single IS1 insertion upstream of *yrfF*.

## Results

### Macrophage-adapted *E. coli* evolved the ability to delay phagosome maturation

**Figure 1:**
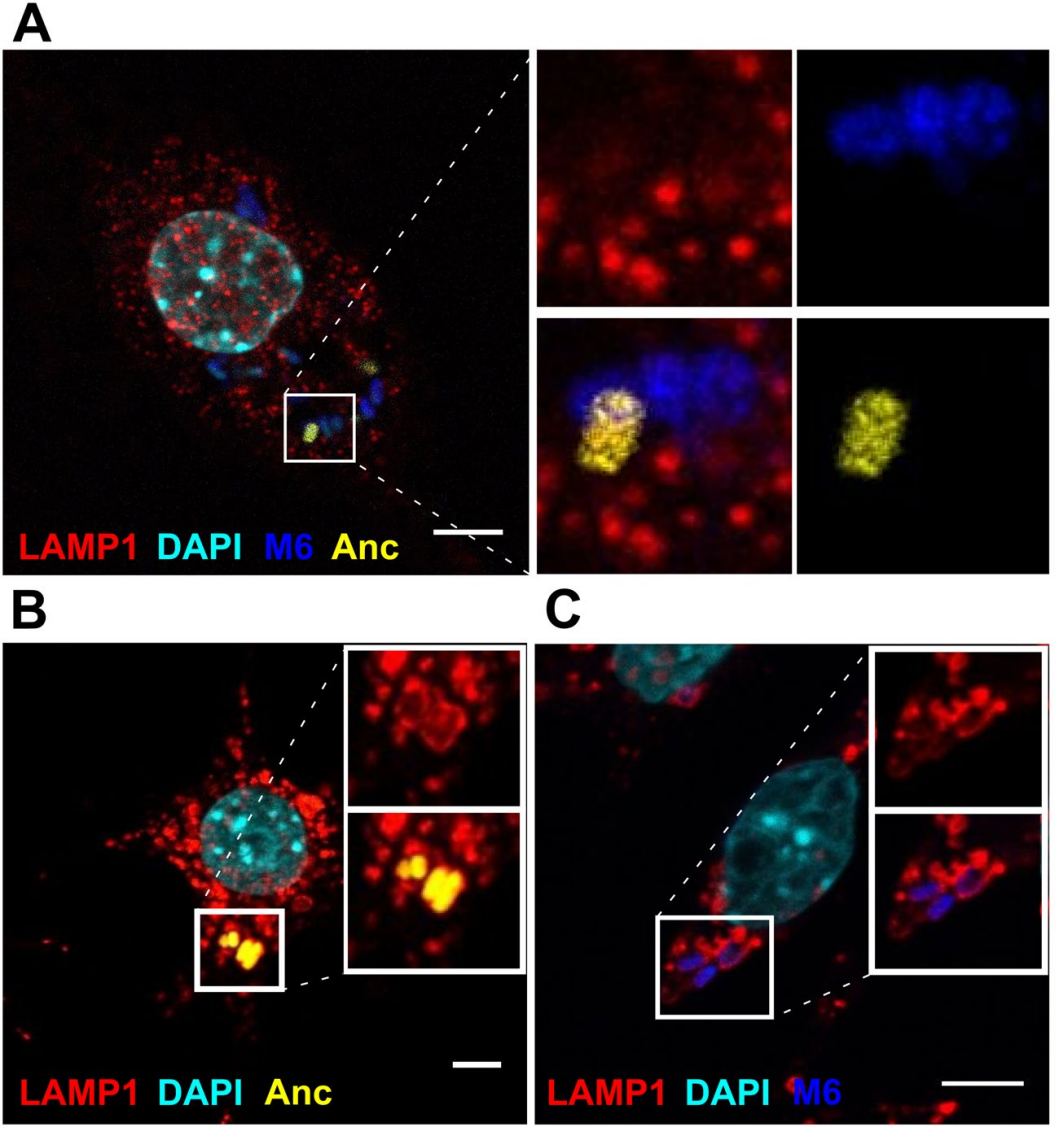
Anc and M6 bacteria-containing phagosomes are negative for the autophagosomal marker LC3 and positive for the late endosomal/lysosomal marker LAMP1. Representative confocal images of infected MΦs. Anc bacteria are shown in yellow and the M6 evolved clone in blue. Scale bar: 5 µm. (**A**) MΦs were infected with a MOI of 20 of Anc and 120 of M6 bacteria. Cells were fixed at 1 h post-infection and stained with α-LC3 antibody (red) and DAPI (cyan). (**B**) MΦs infected with a MOI of 20 of Anc bacteria at 1 h post-infection, fixed and stained with α-LAMP1 antibody (red) and DAPI (cyan). (**C**) MΦs infected with a MOI of 120 of M6 bacteria at 1 h post-infection, fixed and stained with α-LAMP1 antibody (red) and DAPI (cyan).

We have previously isolated mutants of a commensal *E*. *coli* strain that adapted to the presence of MΦs, through an experimental evolution setup^14^. The evolved clones are not only better at evading MΦ phagocytosis *in vitro* but are also more virulent *in vivo* ^14^. Here, we characterize one evolved clone (M6), which contains three new Insertion Sequences of element IS1: one maps to the *yrfF* promoter region, another to the *yiaW* coding region and the third insertion to the *potD* coding region. While the function of the protein encoded by *yrfF* in *E*. *coli* is unknown, its S*almonella* homologue, IgaA is able to repress the RcsCDB regulatory system, which responds to envelope stress and regulates colanic acid capsule synthesis^27^. This is consistent with the mucoid phenotype exhibited by the evolved clone. *yiaW* encodes an inner membrane protein with unknown function, so it is difficult to predict what functional change this mutation could have caused. Finally, the PotD protein of *E*. *coli* is part of the spermidine-preferential uptake system, acting both as the substrate binding and operon repressor protein^28^. We have previously shown that this specific IS1 insertion in the *potD* gene results in increased growth of the bacteria in the presence of high concentrations of the polyamine spermine^14^. On the other hand, and regarding the MΦ cells, polyamines have been described as known inducers of autophagy^29^. Thus, we hypothesised that following an initial acquisition of the insertion in the promoter region of *yrfF*, the occurrence of an insertion in the coding region of *potD* could result in a fitness increase of the bacteria inside MΦs by modulation of their autophagy. To query if this could have happened, we characterized both parental (Anc) and the evolved (M6) bacterial strains in the intramacrophage environment by testing whether they reside in LC3-positive autophagosomes. MΦs were co-infected, as described in the Materials and Methods, with a multiplicity of infection (MOI) of 20 and 120 of Anc and M6 strains, respectively in order to achieve similar numbers of intracellular bacteria. M6 bacteria are able to effectively evade MΦ phagocytosis (Supplementary Fig. S1), hence the need to increase the MOI 6-fold in order to achieve comparable intracellular loads to the Anc strain. Infection was halted after one or three hours, cells were fixed, stained with DAPI and anti-LC3 antibody and imaged by confocal microscopy (Fig. 1A). LC3 staining displayed a punctate cytoplasmic pattern that did not associate with phagosomes containing either Anc or M6 *E*. *coli* (Fig. 1A).

We next asked whether the *E*. *coli*-containing phagosomes bore the late endosomal/lysosomal marker Lysosomal Associated Membrane Protein 1 (LAMP1). For this purpose, activated macrophages were infected with a MOI of either 20 of Anc or 120 of M6 bacteria. MΦs were fixed and stained at one or three hours post-infection (hpi) and imaged by single plane confocal microscopy. Both Anc and M6 could be detected in LAMP1-positive phagosomes (Fig. 1B,C). However, LAMP1 association seemed to be more prominent with phagosomes containing Anc strain, suggesting that evolved bacteria might impair phagosome maturation. Phagosomes mature by progressively becoming more acidic until turning into degradative phagolysosomes through sequential fusion with early endosomes, late endosomes and finally lysosomes. In order to evaluate if Anc and M6 bacteria-containing phagosomes mature at different rates, we quantified the levels of LAMP1 staining by scoring the number of phagosomes containing bacteria in three different categories: completely associated with LAMP1 (LAMP1 staining completely surrounding the bacteria), partially associated (LAMP1 detected only in part of the phagosomal membrane) or not associated (examples can be seen in Supplementary Fig. S2).

**Figure 2:**
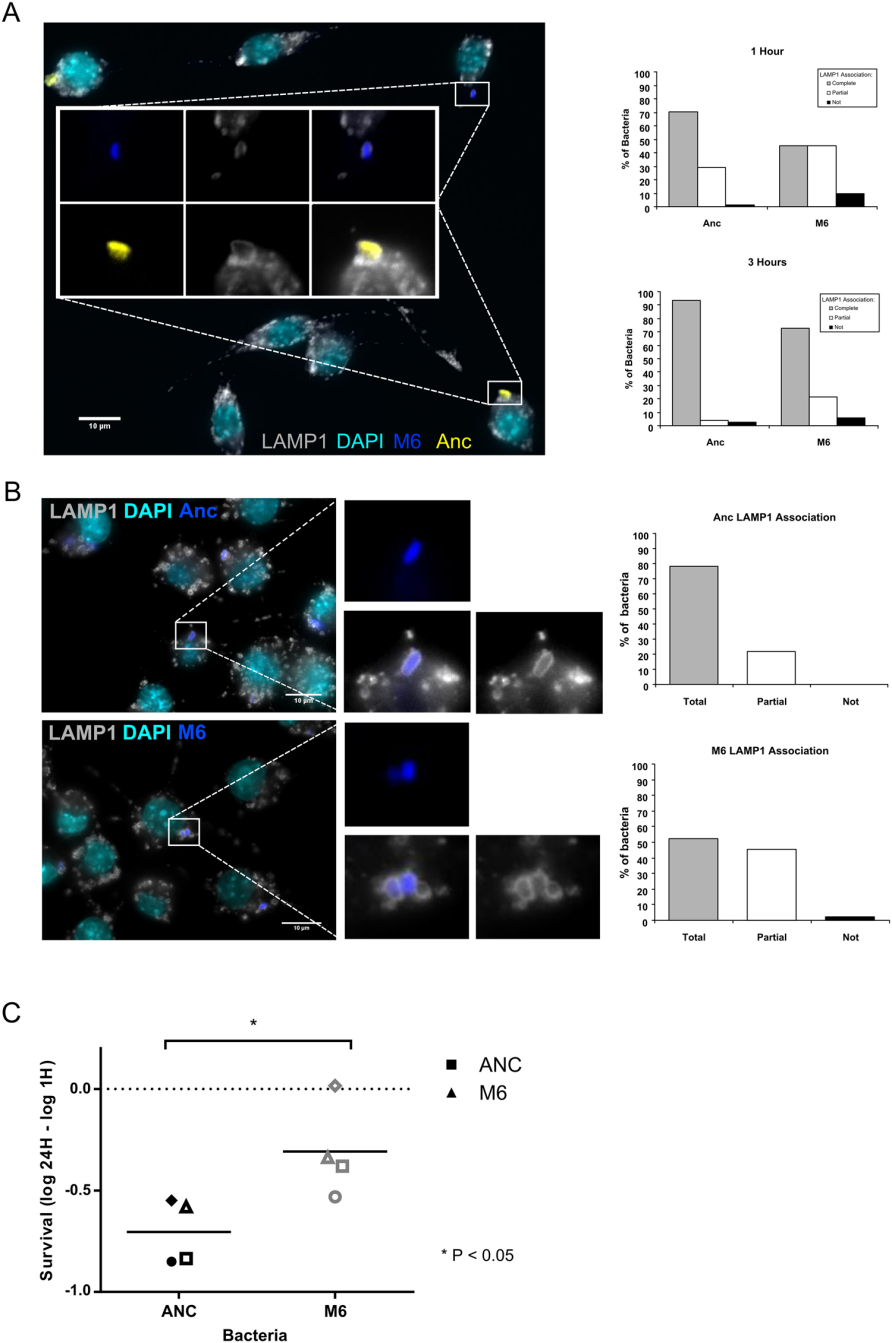
M6-containing phagosomes mature slower and M6 bacteria show enhanced survival inside macrophages. (**A**) Representative fluorescence image of MΦs at 1 h post-infection. Anc bacteria are shown in yellow and the M6 evolved clone in blue. LAMP1 is shown in grey and DNA in cyan. Quantification of LAMP1 association at 1 h and 3 h post-infection. Scale bar: 10 µm. (**B**) Representative fluorescence images of macrophages 1 h post-infection performed in parallel with Anc (top panel) and M6 (lower panel) bacteria. LAMP1 is shown in grey, DNA in cyan and bacteria in blue. Quantification of LAMP1 association at 1 h post-infection is plotted. Scale bars: 10 µm. (**C**) Survival of Anc and M6 bacteria inside macrophages assessed 24 h post-infection. Each symbol represents one experiment and the bar represents the average survival of four independent experiments. * P < 0.05, Mann-Whitney test.

At 1 hpi, differential LAMP1 staining was detected (P < 10^−5^, Fisher’s Exact test), with 70%, 29%, and 1% of Anc bacteria completely, partially or not associated with LAMP1 compared with 45%, 45% and 10% of M6 bacteria completely, partially or not associated with LAMP1, respectively (Fig. 2A). By 3 hpi, the difference between strains remained significant (P < 10^−4^), with over 94% of Anc bacteria completely associated with LAMP1, in contrast with 73% of M6 (Fig. 2A). We also investigated if the delay in phagosome maturation occurred at a higher MOI. In order to do this, we infected MΦs as before with a MOI of 960 M6 and 80 Anc bacteria. At 1 hpi, there were significant differences in the levels of LAMP1 association with 86%, 13% and 1% of Anc bacteria completely, partially or not associated with LAMP1, while M6 association levels were 71%, 24% and 5%, respectively (Supplementary Fig. S3). These results strongly suggest that M6 bacteria impair phagosome maturation. To further elucidate if the delay in phagosome maturation is not due to competition between the two strains but an exclusive property of the MΦ-evolved M6 strain, we performed infections with each of the strains independently. In order to minimize differences in the number of intracellular bacteria per MΦ, only MΦs containing from 3 to 8 intracellular bacteria were analysed. Quantification of LAMP1 association with bacteria-containing phagosomes revealed once again significant differences between the strains. At 1 hpi, 78%, 22% and 0% of Anc and 52%, 45% and 3% of M6 bacteria were completely, partially or not associated with LAMP1, respectively (P < 10^−5^) (Fig. 2B). Similar to the co-infection assay, the single infection assay revealed that the association of M6-containing phagosomes with the late endosome/lysosome marker LAMP1 is significantly reduced in relation to Anc-containing phagosomes.

In order to confirm the delay in the fusion of the M6 bacteria-containing phagosomes with lysosomes, we preloaded lysosomes with Cy5-labelled dextran by pulsing for 1 hour and chasing for 2 hours prior to infection with either Anc or M6 bacteria, as described before. Cells were then fixed at 1 hpi or 3 hpi. We monitored the co-localization of dextran with bacteria, which reflects the fusion of dextran-labeled lysosomes with bacteria-containing phagosomes. Because dextran is a soluble marker, it can mostly be detected juxtaposed with the bacteria, which occupy the entire lumen of the phagosome. This juxtaposition renders the quantification of lysosome-phagosome fusion very difficult. Nevertheless, we were able to detect dextran staining in Anc and M6 bacteria-containing phagosomes at 1 hpi (Supplementary Fig. S4) and 3 hpi. In agreement with the results described above, dextran staining was more readily detected in Anc-containing phagosomes (Supplementary Fig. S4), confirming the phagosome maturation delay detected by immunofluorescence staining of LAMP1.

### *E. coli* triple mutant shows increased survival inside macrophages

Due to the slower maturation of the evolved bacteria-containing phagosomes, we hypothesised that they would have increased intracellular survival when compared with their ancestor. Therefore, we tested this hypothesis using a gentamycin protection assay. Samples were taken at 1 hpi and 24 hpi, and the number of bacteria determined by flow cytometry in the presence of the viability dye propidium iodide. We found that M6 bacteria show a significantly increased survival inside MΦs (Fig. 2C). These results suggest that *E*. *coli* evolved in the presence of MΦs, acquired two important traits: an extracellular advantage through its increased ability to escape MΦ phagocytosis^14^ (Supplementary Fig. S1) and an intracellular advantage through the delay of phagosome maturation, increasing its chances of survival.

### Characterization of gene expression changes at the mutated loci

**Figure 3:**
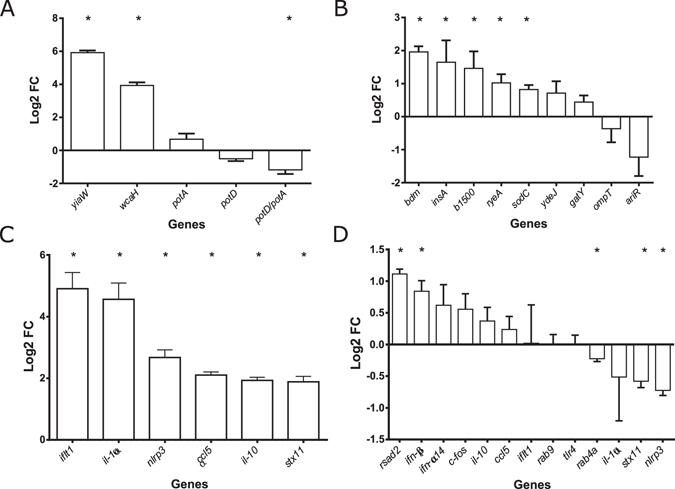
Host and pathogen gene expression at 1 h post-infection. Values represent the average (log2) fold change detected by RT-qPCR from three independent experiments. Error bars represent SEM. (**A**) Expression values of *yiaW*, *wcaH*, *potA*, *potD* and *potD* relative to *potA* in the M6 strain relative to Anc, at 1 h post-infection. (**B**) Confirmation of differential expression of the top 9 differentially expressed transcripts identified by *E*. *coli* Genome 2.0 Array for M6 bacteria relative to Anc 1 h post-infection. (**C**) Confirmation of differential expression of 6 of the 1110 differentially expressed transcripts (Padjusted <0.05) detected by Mouse Gene 2.1 ST Array between infected and uninfected MΦs. (**D**). Relative expression values of 13 out the top 2,881 differentially expressed transcripts (P < 0.05) identified by Mouse Gene 2.1 ST Array between MΦs infected with Anc and M6 strains at 1 h post-infection.

To further characterise the M6 strain, we compared the levels of expression of the genes targeted by the IS1 insertions in the M6 genome, with those of the Anc strain at 1 hpi by RT-qPCR. The observed changes in gene expression are shown in Fig. 3A. Because the *E*. *coli*’s *yrfF* transcript levels were below our detection limit, we tested the expression of *wcaH*, a downstream gene that is negatively regulated by the *yrfF-*encoded protein. *wcaH* expression was significantly increased (P < 0.05, t-test) in the M6 strain. Since *wcaH* encodes a protein involved in the biosynthesis of colanic acid, this is consistent with the colanic acid overproducing phenotype of these bacteria. The *yiaW* transcript levels were significantly upregulated (P < 0.05) in the evolved strain. This is unexpected since the IS1 insertion is in the *yiaW* coding region (263/324nt) and not in its promoter region. However, we cannot be sure if the transcript we are detecting originates from the *yiaW* promoter or from the IS element going in the opposite direction of the *yiaW* mRNA.

Since *potD* is the last gene of the *potABCD* operon and the gene bearing the IS1 insertion, we also tested *potA*, the first gene of this operon, as a proxy of the operon’s transcription rate. Interestingly, the trend of gene expression alteration was the opposite for both genes, with *potA* transcripts being overrepresented and *potD* transcripts underrepresented in the M6 strain in all three replicates. By comparing *potD* with *potA* transcripts levels, we are able to determine the relative amount of the full *potABCD* mRNA in relation to *potA* transcripts and, therefore, in relation to the operon’s transcription level. We found 1.18-fold (log2) less full *potABCD* transcripts in the M6 strain per transcribed *potA* than in the Anc strain (P < 0.05). Thus, we hypothesise that the IS1 insertion decreases *potABCD* mRNA stability, which would explain the significantly lower levels of *potD* relative to *potA* detected for the M6 bacteria (Fig. 3A).

### Genome-wide expression differs in ancestral and evolved bacteria

With the aim of understanding how the M6 MΦ adapted bacteria mediates the phagosome maturation delay described above, we performed a genome-wide gene expression analysis of Anc and M6 bacteria during MΦ infection. Infections were performed three times independently and in parallel with both strains. We then extracted RNA at 1 hpi and the RNA sample was divided for MΦ and bacteria expression arrays.

*E*. *coli* expression arrays revealed significant differences in the gene expression profiles between Anc and M6 strains (Supplementary Table S1) with fold changes (FC) (log2) ranging from 0.55 to −0.36. From the 4371 probe sets, 516 transcripts were found to be differentially expressed at a significance level of P < 0.05 (Empirical Bayes paired test) but only *yiaW* showed a significance of Padjusted <0.05 (Benjamin Hocheberg FDR correction). To further validate the detected changes, we tested the top 10 differentially expressed transcripts, by RT-qPCR.

The FCs detected by RT-qPCR were always higher than the ones detected in the expression arrays. From the 10 transcripts tested, we found differential expression of 6 genes: *b1500*, *bdm*, *insA*, *yiaW*, *ryeA* and *sodC*. This confirms that the M6 bacteria have an altered profile of gene expression (Fig. 3B).

The biofilm-dependent modulation protein encoded by *bdm* is an example of a gene whose expression is positively regulated by the RcsBCD phosphorelay system. *bdm* expression is upregulated upon acid treatment^30^ and has a role in *E*. *coli* adaptation to osmotic stress^31^.

*b1500*, also known as *safA* (sensor associated factor A), encodes a small membrane protein that connects the two-component system EvgS/EvgA and PhoQ/PhoP^32^. *safA* has been shown to contribute to acid resistance mediated by the EvgA/EvgS two-component signal transduction system^32^. This system is also involved in multidrug resistance. On the other hand, the PhoP/PhoQ system responds to external Ca^2+^ and Mg^2+^ levels, regulating transporters and lipopolysaccharide (LPS) modification genes.

*ryeA* is a small RNA controlled by *rpoS* ^33^, which controls *ryeB* (also known as *sdsR*) RNA levels in both *E*. *coli* and *Salmonella* ^33, 34^. *ryeB* interacts with *mutS* mRNA and reduces mismatch repair activity^35^. We found increased expression of *ryeA*, which has been shown to result in decreased levels of *ryeB*. This decrease should lead to increased *mutS* protein levels resulting in increased mismatch repair.

*sodC* encodes a RpoS dependent periplasmatic superoxide dismutase^36–38^. The knock-out of this gene results in increased sensitivity to oxidative stress^38^.

As the phagosome matures, its internal pH drops, the levels of oxidative stress increase and different types of hydrolases, which are able to digest most types of macromolecules, accumulate in its lumen. Several of the genes described (e.g.: *bdm*, acid and osmotic stress; *sodC*, oxidative stress; *ryeA*, increased mismatch repair) can be important for survival in such an environment where the bacteria are under continuous stress and attack by the MΦ.

Interestingly, the IS1 transposable element mRNA (*insA*) is also more abundant in M6 bacteria. This could be due to the increased number of IS1 copies in the M6 genome compared with the Anc genome. However, the number of IS1 elements increased from 7 to 10 copies, *i*.*e*. 1.4-fold, but the transcript levels increased 3.3-fold, strongly suggesting that the *insA* expression is indeed increased. IS1 element encodes two proteins within the same mRNA: the repressor (InsA) and the transposase (InsAB). The transposase is only translated following a ribosomal frameshift event^39^, which makes it impossible to infer transposition rates uniquely based on mRNA expression data. Therefore, the most likely explanation is that one or more of the new IS1 element copies are in a transcriptionally active site, leading to the increased IS1 transcript expression.

These results show that the adaptation of *E*. *coli* to MΦs resulted in changes in its gene expression, consistent with both the observed mutations and the selective pressures it experiences inside MΦs, namely acidic and oxidative stresses.

### Macrophage transcriptome is changed upon infection with evolved bacteria

To query if evolved M6 and its parental Anc strain elicit different responses in MΦs, we performed expression array analyses on Anc, M6 and mock infected MΦs at 1 hpi. As expected^40–44^, MΦ gene expression arrays revealed pronounced differences when comparing infected *vs*. uninfected MΦs with the most differentially expressed gene reaching 5.6 FC (log2). From the 24,479 probes sets, 1,110 transcripts were differentially expressed at a significance level of Padjusted <0.05 (Empirical Bayes, Bejamin Hocheberg FDR correction) and 374 transcripts reached a significance level of Padjusted <0.01, after just 1 hour of contact with *E*. *coli* (Supplementary Table S2). Gene ontology biological process overrepresentation test^45, 46^ of these 374 transcripts revealed a clear overrepresentation of biological processes involved in responses to molecules of bacterial origin and inflammation, *e*.*g*. cellular responses to LPS, NF-kappa β signalling and cytokine biosynthesis (Supplementary Table S3). KEGG pathway analyses^45, 46^ highlighted expression changes in genes involved in Toll-like receptors (TLRs), Nucleotide-binding Oligomerization Domain (NOD) and Mitogen-Activated Protein Kinases (MAPK) signalling pathways (Supplementary Table S4).

We further confirmed, by RT-qPCR, the differential expression of 6 genes (*ccl5*, *ifit1*, *il-10*, *il-1α*, *nlrp3* and *stx11*) with FC (log2) ranging from 1 to 3.2 in the expression arrays. These genes were chosen from the top 374 transcripts because they were also identified as differentially expressed between MΦ infected with Anc *vs.* M6 bacteria. For all genes tested, differential expression between infected and mock-infected cells was confirmed by RT-qPCR with higher FC (average FC increase of 1.65) than detected by the microarrays (Fig. 3C).

Subtler differences were revealed when comparing the transcriptomes of MΦs infected with either Anc or M6 strains. From the 24,479 probe sets, no transcripts reached Padjusted <0.05 significance level and 2,881 transcripts (11.8%) showed a P < 0.05 with changes in expression ranging from 0.15 to 1.17 FC (log2) (Supplementary Table S5). With such reduced changes, we decided to further validate a few genes selected from the group of 2,881 transcripts with P < 0.05 that are involved in MΦ immune response and intracellular trafficking. The transcripts tested were: *ccl5*, *c-fos*, *ifit1*, *ifn-α14*, *ifn-β*, *il-10*, *il-1α*, *nlrp3*, *rab4a*, *rab9*, *rsad2*, *stx11* and *tlr4*. We found that *ifn-β*, *rab4a*, *rsad2*, s*tx11* and *nlrp3* were differentially expressed (P < 0.05, t-test) in MΦs infected with Anc *vs*. evolved M6 (Fig. 3D). The highest change detected, FC (log2) of 1.17 in the arrays and 1.11 by RT-qPCR, was in the expression of the *rsad2* transcript, which is more prevalent in M6 infected MΦs. *rsad2* encodes the interferon inducible antiviral protein viperin. Viperin’s role in antiviral immunity is well documented^47^ but its role in antibacterial immunity is poorly understood. However, it is known that its expression can be stimulated by lipopolisaccharide (LPS) and IFN-β^47^. IFN-β is a type I interferon, whose expression can be triggered by Gram negative bacteria following LPS recognition by TLR4 or CpG DNA recognition by TLR9^48^. As with *rsda2*, *ifn-β* expression is typically induced by viruses and is more prevalent in M6 infected MΦs. IFN-β functions to alert surrounding cells and induce the expression of type I interferon induced genes, which are able to augment cell-autonomous antiviral and antibacterial defences^49^.

The NLRP3 inflammasome is the most versatile and clinically implicated inflammasome. It is activated in response to a variety of pathogen-associated molecular patterns (PAMPs) and danger-associated molecular patterns (DAMPs). A two signal model has been proposed for *nlrp3* activation. The first signal mediates transcriptional activation of *nlrp3* and pro-IL-1β through recognition of ligands for TLRs, IL-1 receptor, NOD2 and/or TNF receptor. The second signal leads to inflammasome oligomerization and activation and can be of a diverse origin, such as ion fluxes, mitochondrial damage, exposure to bacterial toxins, lysosomal destabilization, bacterial mRNA or ROS, amongst others. The active NLRP3 inflammasome activates caspase 1, which processes both pro-IL-1β and pro-IL-18 into their active forms, leading to the secretion of these very potent pro-inflammatory cytokines. *E*. *coli* has previously been shown to induce NLRP3 activation in MΦs^50, 51^. Enterohemorragic *E*. *coli* (EHEC) is able to target NLRP3 inflammasome activation and block IL-1β cytokine production^52^.

*stx11* encodes Syntaxin 11, a Soluble NSF Attachment Protein Receptor (SNARE) that regulates late endosome/lysosome fusion^53^. Therefore, changes in its expression can be the reason for the impaired phagosomal maturation observed for the M6 bacteria.

We hypothesise that the significantly higher volume (around 6x) of M6 bacterial culture added to the MΦs in order to achieve comparable intracellular infection levels, leads to an increased detection of extracellular PAMPs. This stronger activation of TLRs results in increased *ifn-β* and viperin expression levels in the M6 infected MΦs. *nlrp3* expression, on the other hand, can also be dependent on other receptors such as TNF or NOD2^54^. Therefore, it is possible that Anc bacteria trigger these receptors more than the evolved M6 bacteria, whose colanic acid capsule might more effectively hide the bacterial antigens. This reduced recognition might also explain the slower phagosome maturation detected for the M6 bacteria.

### A single IS1 insertion upstream of *yrfF* causes a delay in phagosome maturation

**Figure 4:**
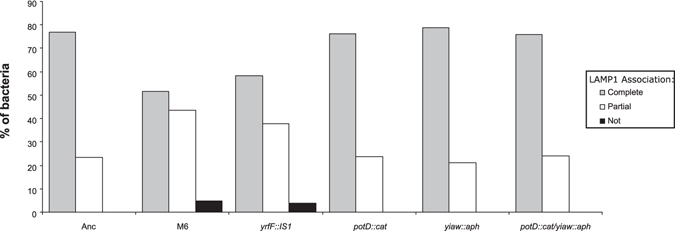
*yrfF* IS1 insertion is sufficient and necessary to cause phagosome maturation delay. Quantification of LAMP1 association with phagosomes containing Anc, M6, *yrfF::IS1*, *potD::cat*, *yiaw::aph* or the double mutant *potD::cat*/*yiaw::aph* at 1 h post-infection.

Since M6 is a triple mutant, we attempted to determine if a particular IS1 insertion alone could cause the phagosome maturation delay. We inserted antibiotic resistance genes into the Anc strain in the same positions where the IS1 insertions are present in the MΦ-adapted M6 bacteria. We inserted a chloramphenicol acetyltransferase expression cassette (*cat*) in *potD* and a kanamycin resistance gene (*aph*) in *yiaW*. Attempts to construct a *yrfF* tetracyclin resistance gene insertion failed, so we sampled clones from the population where M6 originally emerged^14^. We isolated a clone containing the *yrfF* IS1 insertion and lacking the two other insertions in *potD* and *yiaW*. This clone has a mucoid phenotype similar to M6. MΦs were infected in parallel with Anc, M6, *potD::cat*, *yiaw::aph*, the double mutant *potD::cat*/*yiaw::aph* and the *yrfF::IS1* clone as described before. Cells were then fixed at 1 hpi, stained with DAPI and anti-LAMP1 antibody and the phagossomal LAMP1 association quantified as previously (Fig. 4). Neither the *potD::cat*, *yiaw::aph* nor the double mutant *potD::cat*/*yiaw::aph* showed any association differences when compared to the Anc (P > 0.5 for all, Fishers Exact test). Strikingly, the clone carrying only the IS1 *yrfF* mutation showed a significantly reduced association (P < 0.005), just as the M6 triple mutant strain (P < 0.0005). Furthermore, the LAMP1 association detected for the *yrfF* single mutant was not significantly different from what was observed for the M6 bacteria (P > 0.5). These results strongly suggest that the phagosomal maturation delay phenotype is caused by a single IS1 insertion in the regulatory region of *yrfF*.

The IS1 insertion in *yrfF* promoter region is therefore not only responsible for the increased ability to evade MΦ phagocytosis, but also the cause of the slower maturation of the M6-containing phagosome.

## Discussion

Several pathogenic bacteria have evolved to survive and take advantage of the intra-MΦ milieu. In order to do this, they must cope with a hostile environment. During phagosome maturation, luminal pH drops, the levels of oxidative stress rise and the activity of degradative enzymes increases. However, several intracellular pathogens have evolved strategies to subvert this process. *Mycobacterium tuberculosis* (Mtb) and *Legionella pneumophila* are two examples of bacteria that impair phagosome maturation^17, 18, 20, 21^. Moreover, *S*.*flexneri* avoids degradation in phagolysosomes by translocating to the cytoplasm, a strategy that can also be followed by Mtb^19, 22^. Adherent-invasive *E*. *coli* (AIEC) have also acquired the ability to replicate within the phagolysosome. Moreover, as in the case of *Salmonella*, AIEC replication is dependent on phagosomal acidification^55^. In this study, we show that the MΦ-adapted M6 *E*. *coli*, which differs from the commensal K12 *E*. *coli* by only three IS1 insertions, is able to delay the recruitment of the late endosomal/lysosomal marker LAMP1 to the phagosome and shows increased survival inside MΦs. We thus found that the evolution of the commensal K12 strain, when recurrently exposed to MΦs results in the emergence and spread of adapted clones, which carry mutations causing gene expression changes when they enter MΦs and that provide these bacteria the ability to alter the MΦ transcription profile.

Remarkably, we revealed that a single IS1 insertion in the *yrfF* promoter region is responsible for the phagosomal maturation delay. *E*. *coli*’s yrfF, the *Salmonella*’s igaA homologue, is the repressor of the Rcs phosphorelay system, a system involved in controlling biofilm formation, motility, virulence, colanic acid production and remodelling of the cell surface^27^.

We attempted to compare the expression of the *yrfF* mRNA but the low levels of expression did not allow for an accurate quantification by qPCR. For this reason, we tested a gene downstream of the Rcs system, *wcaH*, which encodes the colanic acid biosynthesis protein, and found it to be overexpressed in the M6 bacteria, confirming the altered activation of the Rcs system. The intra-MΦ transcriptome differences between Anc and M6 strains were assessed by microarray analysis and confirmed by RT-qPCR. From the top ten differentially expressed transcripts detected in the arrays, we were able to confirm the differential expression of six, validating our hypothesis that the M6 bacteria have an altered gene expression profile. From the six genes, there are several whose increased expression can translate into beneficial characteristics in the phagosome environment, such as *bdm*, which is upregulated upon acid treatment; *sodC*, which can increase resistance to oxidative stress; or *ryeA*, whose increased expression can enhance mismatch repair^33, 35^. Furthermore, the regulation of most of the differentially expressed genes can be related to the Rcs phosphorelay system as the majority of genes are either directly regulated by it (ex.: *bdm*, *wcaH*) or by stress response regulons/systems that interact with the Rcs system (ex.: PhoP/PhoQ system^56^ (*b1500*), RpoS regulon^57^ (*ryeA* and *sodC*).

We also assessed how the MΦs responded to Anc and M6 strains at 1 hpi. We found a clear response by the MΦs to the presence of bacteria with around 4.5% of transcripts differentially expressed (Padjusted <0.05), but reduced differences when comparing infection with Anc or the M6 strain. This is not surprising for two closely-related bacteria. Indeed, Mavromatis and colleagues performed co-transcriptomics experiments of MΦ infected with two phenotypically different uropathogenic *E*. *coli* strains, one able to survive and another that is unable to survive within MΦs^44^. These authors found a clear transcriptional response to bacterial infection and differential bacterial gene expression programs but could not confirm any host gene expression differences when comparing infection with the different bacteria at the early time points of 2 and 4 hpi^44^. We attempted to confirm, by RT-qPCR, the differential expression of 13 genes and could confirm the differential expression of five of them at 1 hpi. From these five, three are involved in immune response: *rsad2*, *ifn-β* and *nlrp3*. M6 infection induces higher expression of *rsad2* and *ifn-β*, two genes more commonly associated with viral infections^47, 49^. We hypothesise that the increase in *ifn-β* and *rsda2* expression is caused by increased LPS recognition that results from the significant higher volume of bacteria culture necessary to achieve comparable numbers of intracellular bacteria. On the other hand, *nlrp3* expression is increased in Anc relative to M6 bacteria. This transcript has been shown to be targeted for downregulation by pathogenic *E*. *coli*, demonstrating its importance in bacterial infection^52^. We postulate that M6 bacteria are being detected differently from the Anc and hence the differential expression of *nlrp3*. Rab4a and Syntaxin 11 are both involved in the regulation of membrane trafficking: Rab4a regulates early endosome sorting and recycling^58^ and Syntaxin 11 late endosome-to-lysosome trafficking^53^. Therefore, it is tempting to speculate that the lower levels of Syntaxin 11 are the cause of the phagosome maturation delay observed for the M6-containing phagosomes.

In summary, we show here that an *E coli* clone isolated after 30 days of co-culture with MΦs evolved not only the ability to evade MΦ phagocytosis but also shows increased survival inside MΦ due to the subversion of phagosome maturation. The three new IS insertions detected in M6 bacteria induce an altered gene expression profile, which results in altered recognition by MΦs. Increased M6 survival can be attributed to either one or both reasons: the altered gene expression profile of M6 bacteria by itself results in increased survival and/or the different detection by the MΦ allows M6 bacteria to go through a less stressful phagosomal environment, which results in the increased survival of this strain. Overall, our results indicate that the use of bacterial experimental evolution under important host-associated selective pressures, together with genomic and phenotypic analysis can unravel important strategies underlying host-microbe interactions.

## Materials and Methods

### Strains and Media

The RAW 264.7 murine MΦ cell line was maintained in an atmosphere containing 5% CO_2_ at 37 °C in RPMI 1640 (Gibco) supplemented with 2 mM L-glutamine (Invitrogen), 1 mM sodium pyruvate (Invitrogen), 10 mM Hepes (Invitrogen), 100 U/ml penicillin/streptomycin (Gibco), 50 µM 2-mercaptoethanol solution (Gibco), 50 µg/ml gentamicin (Sigma) and 10% heat-inactivated fetal calf serum (FCS, standard RPMI complete medium). Infections were performed in RPMI-Strep medium, which is similar to standard RPMI complete medium but contains streptomycin (100 µg/ml) as the sole antibiotic.

Three different strains of *Escherichia coli* were used, two were the reference/Ancestral strain: MC4100-YFP and MC4100-CFP (MC4100, galK::CFP/YFP, AmpR StrepR), which contain the yellow (YFP) and cyan (CFP) alleles of GFP integrated at the galK locus in MC4100 (*E*.*coli* Genetic Stock Center #6152) and differ only by *yfp*/*cfp* locus that is constitutively expressed^59^. The third strain is M6, a clone of MC4100-CFP that was evolved in the presence of MΦs for 450 generations^14^. This strain has three new IS1 element insertions, in the coding regions of *potD* (1,084,946 nt), *yiaW* (3,640,515 nt) and in the regulatory region of the *yrfF* (3,411,601 nt) gene, as described elsewhere^14^. Bacteria were grown in RPMI-Strep in similar conditions to the MΦ cell line.

### Immunofluorescence

Two days before the experiments, 2 × 10^5^ MΦs were seeded per 24 well plate on coverslips in RPMI-Strep. The day before the experiments, MΦs were activated with 2 µg/ml of CpG (5′TCCATGACGTTCCTGACGTT3′, Sigma) in RPMI-Strep for 24 h. This activation allows for an increased number of phagocytosed bacteria^14^. Infection was performed by adding RPMI-Strep containing bacteria and spinning for 5 min. at 182x *g*. Thirty min. post-infection, MΦs were extensively washed with warm PBS and observed under the microscope to confirm effective removal of extracellular bacteria. PBS was then replaced by warm RPMI-Strep. At the specified time points, coverslips were removed, washed in PBS and fixed with 4% paraformaldehyde (PFA) in PBS for 15 min. at RT. When α-LC3B antibody (Cell Signalling #2775) was used, cells were further treated with cold (−20 °C) methanol for 5 min. at RT. Cells were blocked and permeabilized by incubation with 1% BSA (Sigma), 0.05% saponin (Sigma) and 1% fetal calf serum (FCS, Gibco/Invitrogen) in PBS (P/B sol.) for 30 min. at RT. Primary antibody stain was performed in the P/B sol. at 4 °C O/N. Anti-LC3B (Cell Signalling #2775) and anti-LAMP1 antibodies (Hybridoma bank 1D4B-c) were both used at 1/500 dilution. Following primary antibody staining, coverslips were washed in PBS prior to incubation in P/B sol. containing the specific Alexa fluor-labelled secondary antibodies (Invitrogen) at a dilution of 1:1000.

### Dextran lysosomal loading

MΦs were incubated for 1 h with 50 µg/ml Cy5-labelled dextran (MW 10 kDa) at 37 °C and washed 3 times with warm RPMI-Strep. Cells were incubated for further 2 h prior to infection in order to allow dextran to accumulate in the lysosomes. In the case of dextran-labelled cells, the fixation period was extended to 30 min. with 4% PFA in PBS at RT.

### Insertion mutants

Mutants were constructed using the red swap methodology^60–62^, by which antibiotic resistance cassettes were inserted in the same positions as the IS1 insertions were found in the M6 strain. Briefly, primers to amplify resistance expression cassettes were designed containing 50 base flanking regions homologous to the targeted genomic region: *potD* (1,084,946 nt) or *yiaW* (3,640,515 nt) in order to mediate the correct insertion of the foreign DNA (Supplementary Table S6). Chloramphenicol (*cat*) and Kanamycin (*aph*) resistance cassettes were amplified from the plasmids pKD3^60^ and pCR2.1 (Invitrogen), respectively. The PCR products were electroporated into Anc bacteria previously transformed with pKD46 (red recombination helper plasmid). Antibiotic resistant colonies were selected (Chloramphenicol or Kanamycin) and the correct insertion of the fragments confirmed by PCR. In order to avoid any unwanted genomic rearrangements caused by the red recombinases, the construct was transferred by P1 transduction to an independent Anc-CFP clone. As before, insertion mutants were selected by their antibiotic resistance and the insertion sites reconfirmed by PCR. A double insertion mutant *potD::cat*/*yiaW*::*aph* was constructed by sequential P1 transductions and double antibiotic selection. All mutant cultures were tested for phage contamination with negative results. The *yrfF::IS1* clone was selected from day 25 of the 6^th^ line of the original MΦ evolution experiment^14^ by picking mucoid colonies and selecting a colony positive for *yrfF* IS1 insertion and negative for *yiaW* and *potD* IS1 insertions by PCR.

### Microscopy

Confocal single plane or Z-stacks were acquired on a Leica SP5 confocal, using a 63 × 1.3NA oil immersion objective, using HyD detectors in Standard Mode.

Widefield micrographs were acquired on a Leica DMRA2 upright microscope, equipped with a CoolSNAP HQ CCD camera, using the 100 × 1.4NA oil immersion objective, DAPI + Cy5 fluorescence filter sets and DIC optics, controlled with the MetaMorph V7.5.1 software. All images were analysed in FIJI software^63^.

LAMP1 staining was quantified by visualization of 0.4 µm-step Z-stacks throughout the full depth of the bacteria and classified into three categories: completely associated, partially associated or not at all associated with intracellular bacteria (Supplementary Fig. S2).

### Flow Cytometry

The number of bacteria was quantified prior to infection with a LSR Fortessa Flow Cytometer using a 96 well plate auto sampler. Samples were always ran in the presence of SPHERO (AccuCount 2.0 µm blank particles) in order to accurately quantify bacterial cultures. Briefly, flow cytomery samples consisted of 180 µl of PBS, 10 µl of SHERO beads and 10 µl of a 10^−2^ dilution of the bacteria culture in PBS. Bacteria concentration was calculated based on the known number of beads added.

CFP was excited with a 442 nm laser and measured with a 470/20 nm pass filter. YFP was excited using a 488 nm laser and measured using a 530/30 nm pass filter.

### Intracellular survival assays

MΦs were seeded in 24 well plates at 1 × 10^6^ per plate and treated/infected as described above. Following the PBS wash (30 min. post-infection), MΦs were incubated with RPMI-Strep/Gent (containing 100 µg/ml of Streptomycin and Gentamycin). Samples were collected at the specified time points by scraping the wells, followed by washing 3x with PBS to eliminate any extracellular bacteria. MΦs were lysed and the bacteria released by spinning for 5 min. at 10,600x *g*. Ten µl of the 500 µl sample were used for FACS analyses. All samples were ran in the presence of the viability dye propidium iodide (20 µM). For each experiment, at least three wells per condition per time point were performed.

### RNA extraction and purification

MΦs were seeded in 6 well plates at 2.4 × 10^6^ per plate and treated as described for IF experiments with the unique difference that all washes were performed with warm RPMI-Strep. At 1 h post-infection, MΦs were again repeatedly washed with warm RPMI-Strep prior to RNA extraction, which was performed using the Direct-zol RNA MiniPrep Kit (Zymo research), according to the manufacturer’s specifications, including in column DNAse treatment for 20 min.

### Microarrays

The concentration and quality of all RNA samples was determined using a nanodrop 2000 spectrophotometer (Thermo Scientific) and confirmed by using a Bioanalyser 2100 with a RNA 6000 Nano Assay (Agilent Technologies, Palo Alto, CA). Only samples with RIN over 8 were used for gene expression analyses. RNA was processed for use on Affymetrix (Santa Clara, CA, USA) Mouse Gene 2.1 ST Array by using the Ambion WT Expression Kit (Life Technologies, CA, USA) and Affymetrix GeneChip WT Terminal Labeling Kit, according to the manufacturer’s protocols. For *E*. *coli* gene expression analysis, 25 µg of infected MΦ RNA were enriched in bacterial mRNAs with MICROBEnrich kit (Ambion) and cleaned with MEGAclear transcription clean-up kit (Ambion) in accordance with the manufacturer’s instructions. RNA was processed for use on Affymetrix (Santa Clara, CA, USA) *E*. *Coli* Genome 2.0 Array by using pico profiling protocol of Whole Transcriptome Amplification Kit (WTA2), according to the manufacturer’s protocols. mRNA amplification, labelling, and all array procedures were performed by Instituto Gulbenkian de Ciência Gene Expression Unit (IGC, Oeiras, Portugal). Cel files containing raw data were processed using chipster with standard RMA normalization.

### Quantitative PCR

RNA was treated with RQ1 DNase (Promega), according to the manufacturer’s protocol. Reverse-transcriptase reaction was performed with M-MLV RT (Promega) or Superscript IV (Thermo Fisher Scientific) using random primers (Promega) according to the manufacturer’s instructions.

qPCR was performed in BioRad CFX 384 with iTaq Universal SYBR Green Supermix (BioRad). MΦ and *E*. *coli* cDNAs were diluted 10- and 20-fold, respectively when reverse-transcribed with M-MLV RT or 100-fold when transcribed with Superscript IV before being used for qPCR. The cycling conditions were as follows: one step of 5 min. at 95 °C and then 40 cycles of 30 sec. at 95 °C, 30 sec. at 59 °C (60 °C for murine cDNA) and finally 30 sec. at 72 °C. Melt curve analysis was performed to verify product homogeneity. All reactions included three replicates for each sample. We used a relative quantification method of analysis with normalization against a reference gene. Data were normalized by the Pfaffl method^64^ using *hfq* and *actinB* housekeeping genes as references for *E*. *coli* and murine cDNA, respectively. All primers used in this study are listed in Supplementary Table S6.

### Statistical Analysis

LAMP1 staining quantifications were analyzed using Fisher exact test on a 2 × 3 contingency table. For bacterial survival inside MΦs a Mann-Whitney test was performed. For qPCR data, a t-test was performed on fold change values (log2). *E*. *coli* expression arrays data was analysed in R software (R core team 2015) library Limma^65^ using an empirical Bayes paired test. MΦ expression arrays of Anc *vs*. M6 infected MΦ were analysed in R software (R core team 2015) library Limma^65^ using an empirical Bayes paired test. MΦ expression array data comparing infected *vs*. uninfected cells was performed with chipster software^66^ using empirical bayes statistical test. Padjusted values were calculated using Benjamin Hocheberg FDR correction.

## Electronic supplementary material

Supplementary Figures

Dataset TableS1, Dataset TableS2, Dataset TableS3, Dataset TableS4,Dataset TableS5,Dataset TableS6
