# Supplementary material for: Commensal-to-pathogen transition: One-single transposon insertion results in two pathoadaptive traits in Escherichia coli -macrophage interaction
Source: Sci Rep. 2017 Jul 3;7:4504. doi: 10.1038/s41598-017-04081-1 (PMC5495878; doi:10.1038/s41598-017-04081-1)
Supplement: Supplementary file 1 — Supplementary Figures [file 41598_2017_4081_MOESM1_ESM.pdf]

Commensal-to-pathogen transition: One-single transposon insertion results in two pathoadaptive traits in *Escherichia coli* -macrophage interaction.

João T. Proença[1], Duarte C. Barral[2,]* and Isabel Gordo[1,]*

[1]Instituto Gulbenkian de Ciência

[2]CEDOC, NOVA Medical School|Faculdade de Ciências Médicas, Universidade NOVA de Lisboa

*corresponding author: igordo@igc.gulbenkian.pt, duarte.barral@nms.unl.pt

Supplementary Figures

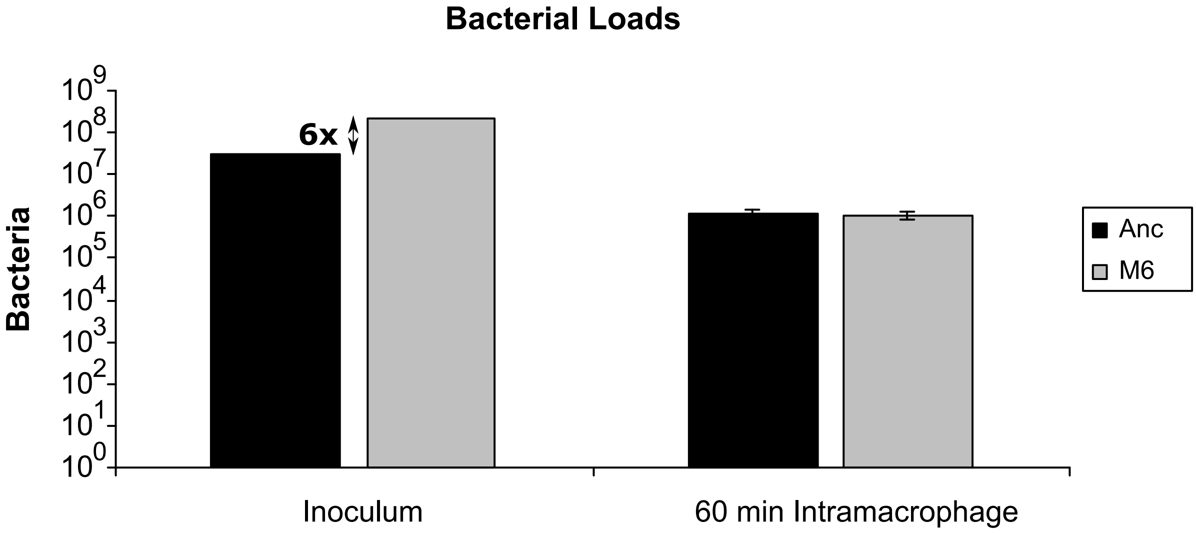

Supplementary Fig. S1 –**Quantification of intracellular M6 and Anc bacteria.**

Infection of $3x10^5$ MΦs with MOI of 50 for the Anc bacteria and 300 for the M6 bacteria resulted in the detection of similar numbers of intracellular bacteria.

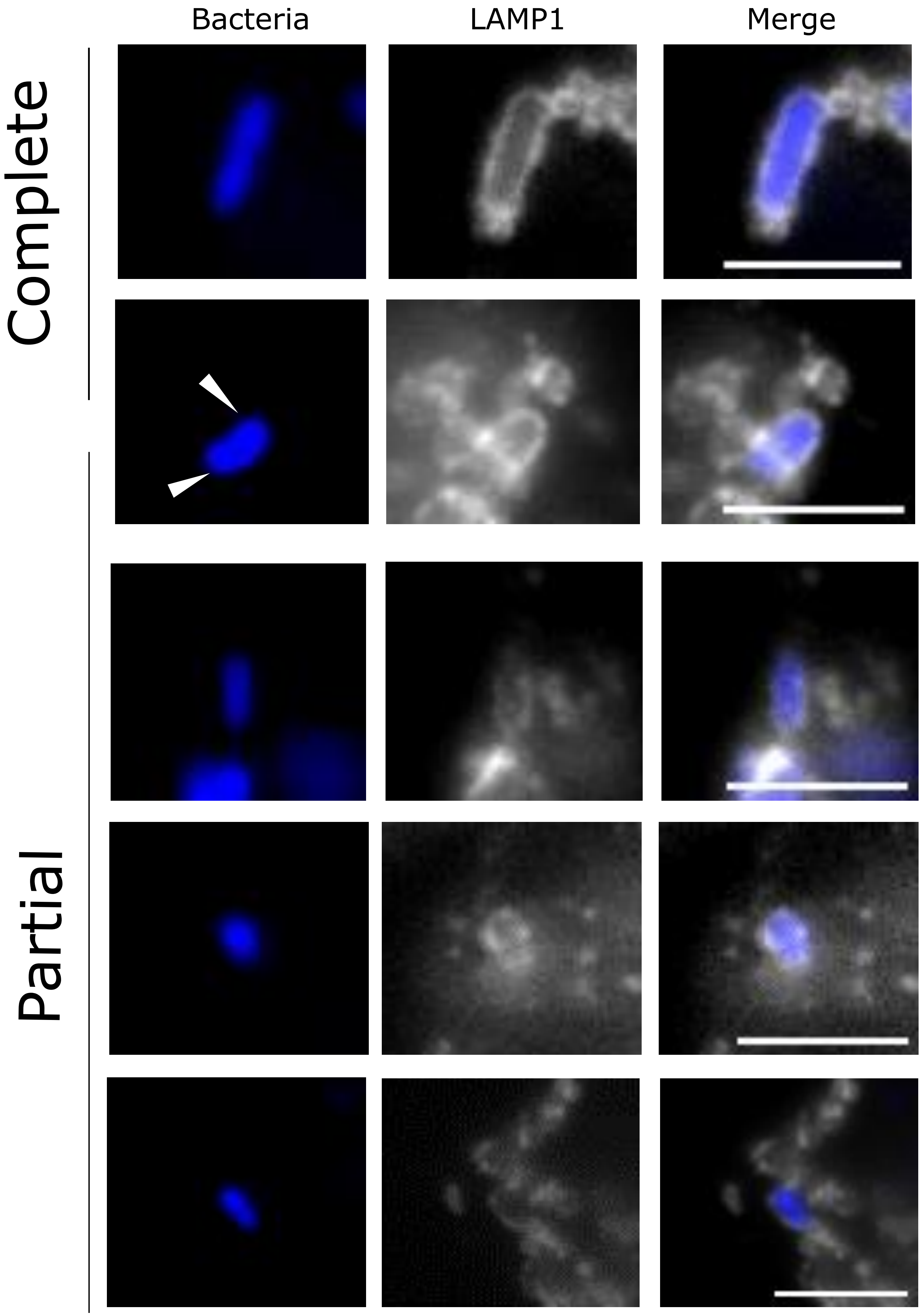

Supplementary Fig. S2 –**Representative images and their classification in terms of LAMP1 association.**

Images were taken 1 h post-infection with Anc strain . LAMP1 is shown in grey, DAPI in cyan and Anc bacteria in blue. Scale bar: 5 μm.

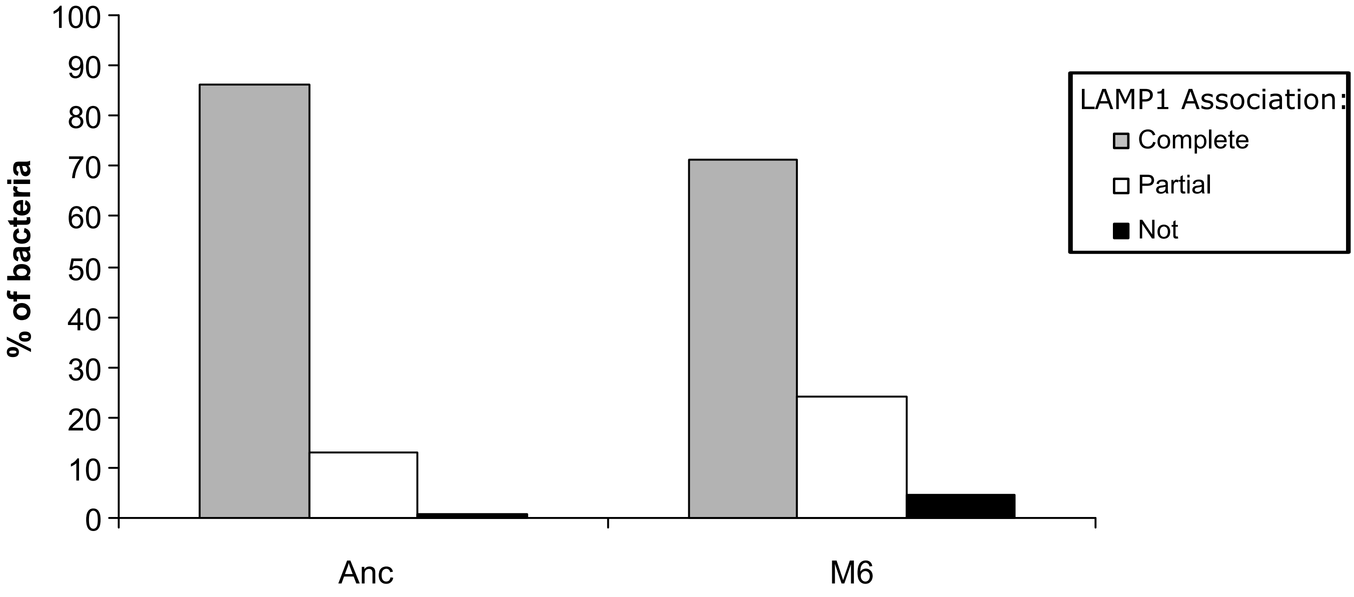

Supplementary Fig. S3 –**Quantification of LAMP1 association in MΦs infected at a high MOI.** MΦs infected with a MOI of 960 for M6 and 80 for the Anc strain at 1 h post-infection revealed significantly different patterns of LAMP1 staining (P<0.001, Fisher exact test).

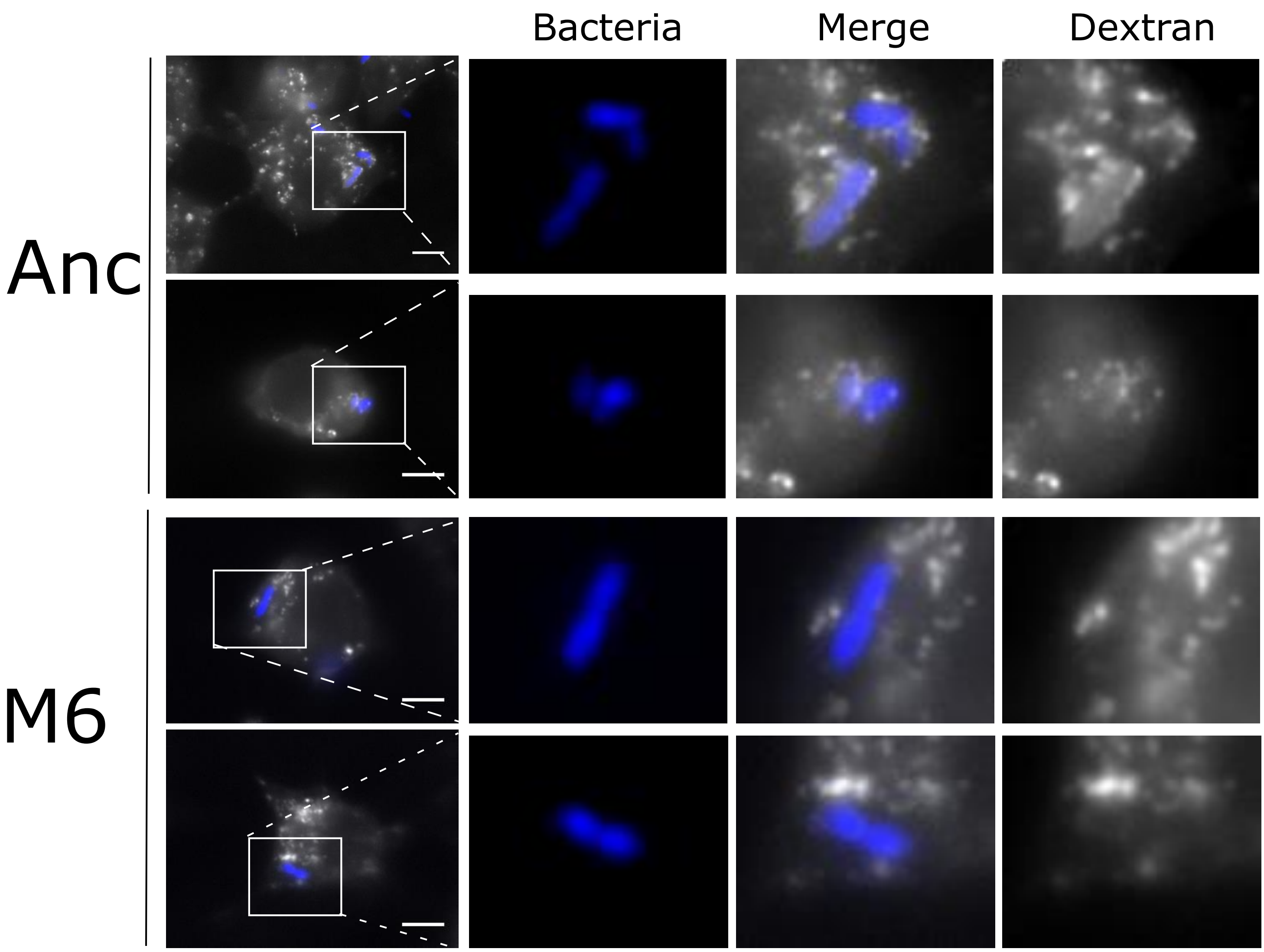

Supplementary Fig. S4 –**Dextran-labelled lysosomes are readily detected in Anc-containing phagosomes at 1h post infection.**

Representative fluorescent images of MΦs labelled with dextran at 1 h post-infection with Anc or M6 bacteria. Bacteria are represented in blue and dextran is represented in grey. Scale bar: 5 µm.
